# Prospective clinical evaluation of a novel anatomic cuff for forearm crutches in patients with osteoarthritis

**DOI:** 10.1186/s12891-017-1459-7

**Published:** 2017-03-14

**Authors:** Thomas Hügle, Ansgar Arnieri, Margerita Bünter, Stefan Schären, Annegret Mündermann

**Affiliations:** 1 Department of Rheumatology, University Hospital Basel, Basel, Switzerland; 2 Department of Rheumatology, Centre Hospitalier Universitaire Vaudois and University of Lausanne, Lausanne, Switzerland; 3 Clinic for Orthopaedics and Traumatology, University Hospital Basel, Basel, Switzerland; 4 Department of Spinal Surgery, University Hospital Basel, Basel, Switzerland; 5 Department of Biomedical Engineering, University of Basel, Basel, Switzerland

**Keywords:** Crutches, Walking, Pain, Comfort, Crutch design

## Abstract

**Background:**

The use of forearm crutches has been associated with pain and neuropraxia along the ulnar bone. Whilst anatomic grips have improved comfort of crutch walking, to date anatomic forearm cuffs have not been clinically evaluated. The aim of this clinical pilot study was to determine if the use of forearm crutches with anatomic cuffs reduces pain and increases comfort and function in long-term users of forearm crutches during a 4-week period.

**Method:**

Prospective study in ten patients suffering from end-stage osteoarthritis of the lower extremity. All participants were long-term users of conventional forearm crutches. Participants used forearm crutches with an anatomically shaped cuff for 4-weeks. General health was assessed using the SF-36, and the crutches were evaluated using a newly developed questionnaire focusing on symptoms along the forearm.

**Results:**

Pain and paresthesia along the forearms decreased by 3.3 points (95% confidence interval difference (CI): [−5.0; −1.6], *p* = .004) and 3.5 points (95%CI: [−5.1; −1.9], *p* = .002), respectively, after using the crutches with the new anatomic cuff for 4 weeks. Comfort and sense of security of crutch use increased by 3.0 points (95%CI: [1.3; 4.7], *p* = .007) and 2.4 points (95%CI: [0.7; 4.1], *p* = .024). Cross-correlation analysis revealed correlations among items in the same item category and no correlations between items of different item categories of the new questionnaires.

**Conclusion:**

An anatomically shaped cuff increases comfort of forearm crutches. Further research should confirm long-term clinical improvement.

**Trial registration:**

This study was registered retrospectively in ISRCTN (TRN: ISRCTN 11135150) on 14/02/2017.

## Background

Because of known demographic changes, the number of patients requiring permanent walking aids will substantially increase in the near future. Today, more than half a million patients in the USA use crutches permanently, mainly because of chronic musculoskeletal or neurological disorders [1, 2]. In the elderly, mobility is considered a cornerstone of healthy ageing with a reduction of mobility often leading to an overall health decline [3]. The use of walking aids for instance in patients with knee osteoarthritis (OA) significantly reduces pain and improves function and general health [4]. However, crutch walking requires twice the energy compared to normal gait [5, 6] especially when the patient adopts an asymmetric walking pattern. This can frequently lead to overuse symptoms of the upper extremity especially with forearm crutches [7].

While in the USA axillary crutches are predominantly used, conventional forearm crutches are more common in Europe. The advantages of forearm crutches are the absence of pressure on the axilla with potentially associated nerve damage [8] and their lighter weight [9]. Because of the greater load on the hands and forearm with forearm crutches, anatomic handles have been developed to increase the size of the contact area and reduce the pressure between the hand and the handle [10]. Besides pain in the hands, local overuse conditions along the forearms such as hematoma and skin bruises are frequently observed after prolonged crutch use. This is not surprising because around one third of the load during crutch walking is absorbed by the forearm [5, 11]. The ulna is only covered by limited soft tissue, and hence not well protected against pressure and shear forces. Ulnar neuropraxia at the wrist [12] and at the forearm [13] have been described and even ulnar fractures have been reported [14].

**Fig. 1:**
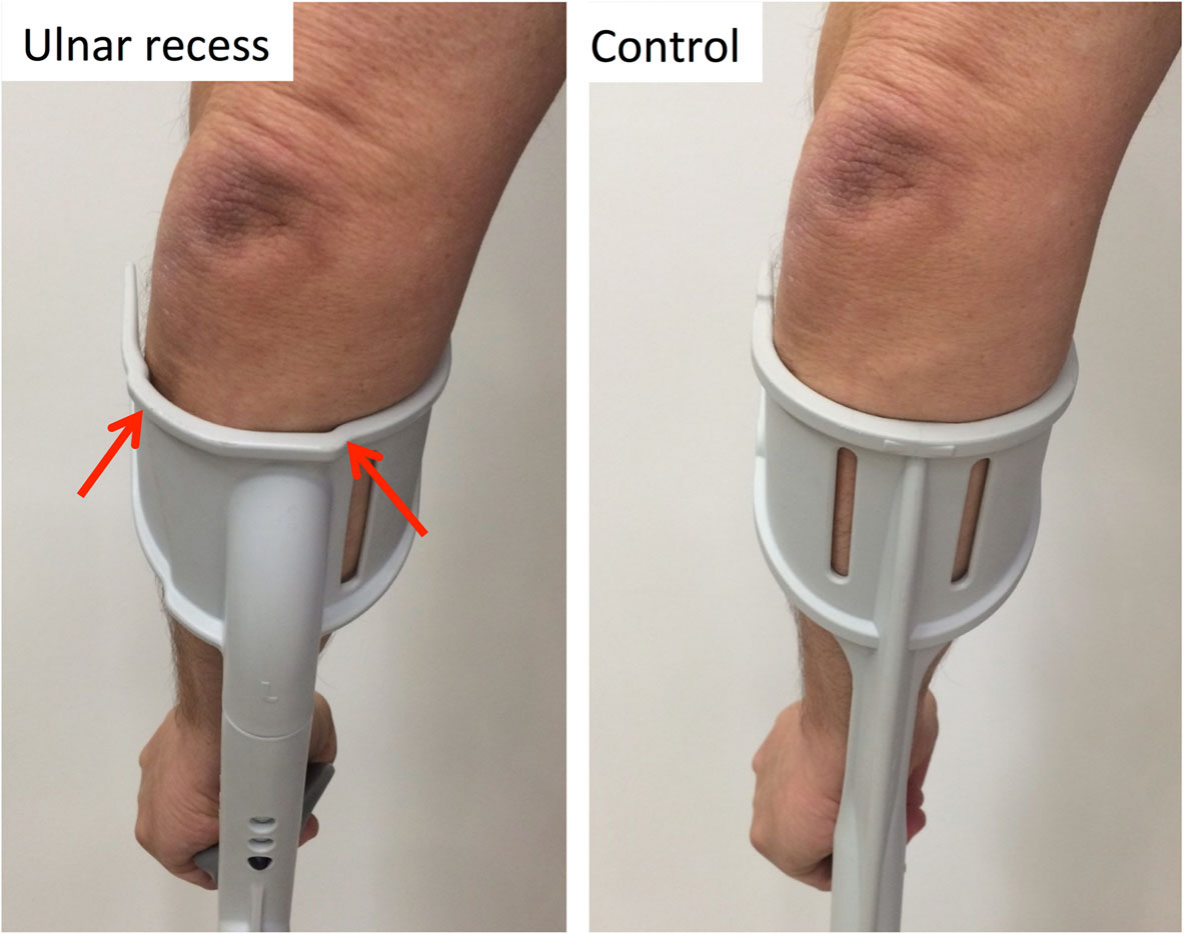
Photograph of the cuff with the lateral recess and a conventional cuff

The main pressure between the forearm and conventional crutch cuff is located over the ulnar bone during crutch walking [15]. With increasing weight-bearing on the crutches during stance, the peak pressure shifts towards the ulnar quadrant suggesting that not only pressure but also shear forces may characterize the crutch-forearm interface. Moreover, this interface may depend on the positioning and orientation of the crutches relative to the arm and body such as crutch abduction [15]. An anatomic cuff for forearm crutches has been developed with the goal of protecting the ulnar bone and distributing the load primarily away from the ulnar bone and well-innervated periosteum and towards surrounding muscles and soft tissue [16]. Figure 1 shows a photograph of the cuff with the lateral recess and a conventional cuff. Initial biomechanical analyses showed lower peak pressures at the ulnar were overuse symptoms are typically located and that the pressure appears to be spread more evenly [16]. The purpose of this clinical pilot study was to determine if the use of forearm crutches with anatomic cuffs reduces pain and increases comfort and function in long-term users of forearm crutches during a 4-week period.

## Methods

### Study design and patients

Ten patients (2 female; mean [range], age: 63.5 [40–80] years; height: 169 [157–188] cm; body mass: 86.5 [74–124]) with different musculoskeletal disorders of the spine or lower extremity (Table 1) participated in this prospective uncontrolled experimental pilot study with a 4-week follow-up period. Only patients who had used bilateral forearm crutches for at least 8 weeks prior were included in this study. All patients who were asked to participate completed the study (no drop outs). Exclusion criteria were infections of hands or forearms, amputation, neuropathy (e.g. due to diabetes or syringomyely), active rheumatic diseases or upper limp injuries. On average, patients had used forearm crutches for 4.5 years, and forearm girth ranged from 24 to 32.5 cm (Table 1). Two patients used crutches with anatomic grips. All participants provided informed consent prior to participation. The study was approved by the local ethics board and conducted in accordance with the Declaration of Helsinki. Written informed consent was obtained from all patients prior to participation.

**Table 1:** Anthropometric and clinical parameters of the study participants

No.	BMI	Forearm girth	Time of crutch use	Condition/reason for crutch use
	kg/m^2^	right/left cm	years	
1	29.7	24.0/24.5	2.5	chronical lumbovertebral syndrom, status post lapidus- arthrodesis/calcaneus-osteotomy
2	32.0	25.5/25.5	2.0	pangonarthrosis bilateral, spinal canal stenosis lumbal
3	27.0	26.0/26.5	1.1	calcaneo-cubiodal arthrosis left
4	28.7	28.5/29.0	6.5	status post calcaneo-talar arthrodesis right
5	31.4	32.0/32.5	7.0	status post tibio-talar arthrodesis right
6	24.8	26.0/25.6	7.0	hip totalendoprosthesis right, status post pertrochanteric femur fractur left, gluteal insufficience
7	35.1	29.0/28.0	2.0	status post hip totalendoprosthesis left.
8	25.4	24.3/24.5	0.5	status post triplearthrodesis right
9	34.0	26.5/26.5	3.0	status post hip totalendoprosthesis right
10	35.7	31.5/28.5	6.0	status post hip totalendoprosthesis bilateral spinal canal stenosis

*BMI* body-mass-index

At baseline, patients received forearm crutches with the anatomic cuff (Ulnar Pro®, Rebotec, Quakenbrück, Germany) with anatomic hand grip (model soft, Rebotec, Quakenbrück, Germany). The length of the crutches were adjusted by the study team to the patients’ hand height during stance with the arms positioned at 20 to 30° elbow flexion [17]. Patients were asked to use the study crutches for 4 weeks. Clinical data were collected at baseline and at 4-weeks follow-up using questionnaires.

### Clinical evaluation

**Fig. 2:**
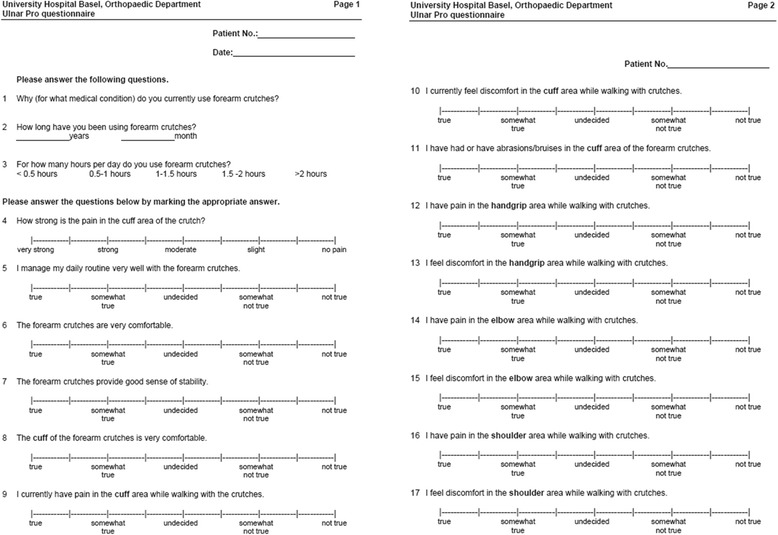
UlnarPro Questionnaire. A specific questionnaire focusing on symptoms along the forearm with a unipolar 9-point Likert-scale. Questions were classified into four different item categories: pain and dysaesthesia along the forearm; comfort and sense of security; symptoms of the hands; symptoms of the shoulders

We developed a questionnaire focusing on symptoms along the forearm (e.g. pain, paresthesia and comfort) with a unipolar 9-point Likert-scale [18] (Fig. 2). Questions were classified into four item categories: pain and dysaesthesia along the forearm; comfort and sense of security; symptoms of the hands; symptoms of the shoulders. Patients also completed the general health questionnaire SF36v2 [19, 20]. Primary endpoint of this study was pain along the forearm. Secondary endpoints were comfort and physical components of the SF36 questionnaire.

### Statistical analysis

All statistical analyses were carried out in GraphPad Prism Version 6 (GraphPad Software, Inc., La Jolla, CA). The data were tested for normality using Shapiro-Wilk tests [21, 22] and D’Agostino-Pearson tests [23]. Significant changes in scores from baseline to follow-up were detected using paired Student’s t-tests. Cross-correlations were calculated to detect significant relationship among scores of the crutch questionnaire. The level of significance for all statistical tests was set a priori to .05.

## Results

### Pain

**Fig. 3:**
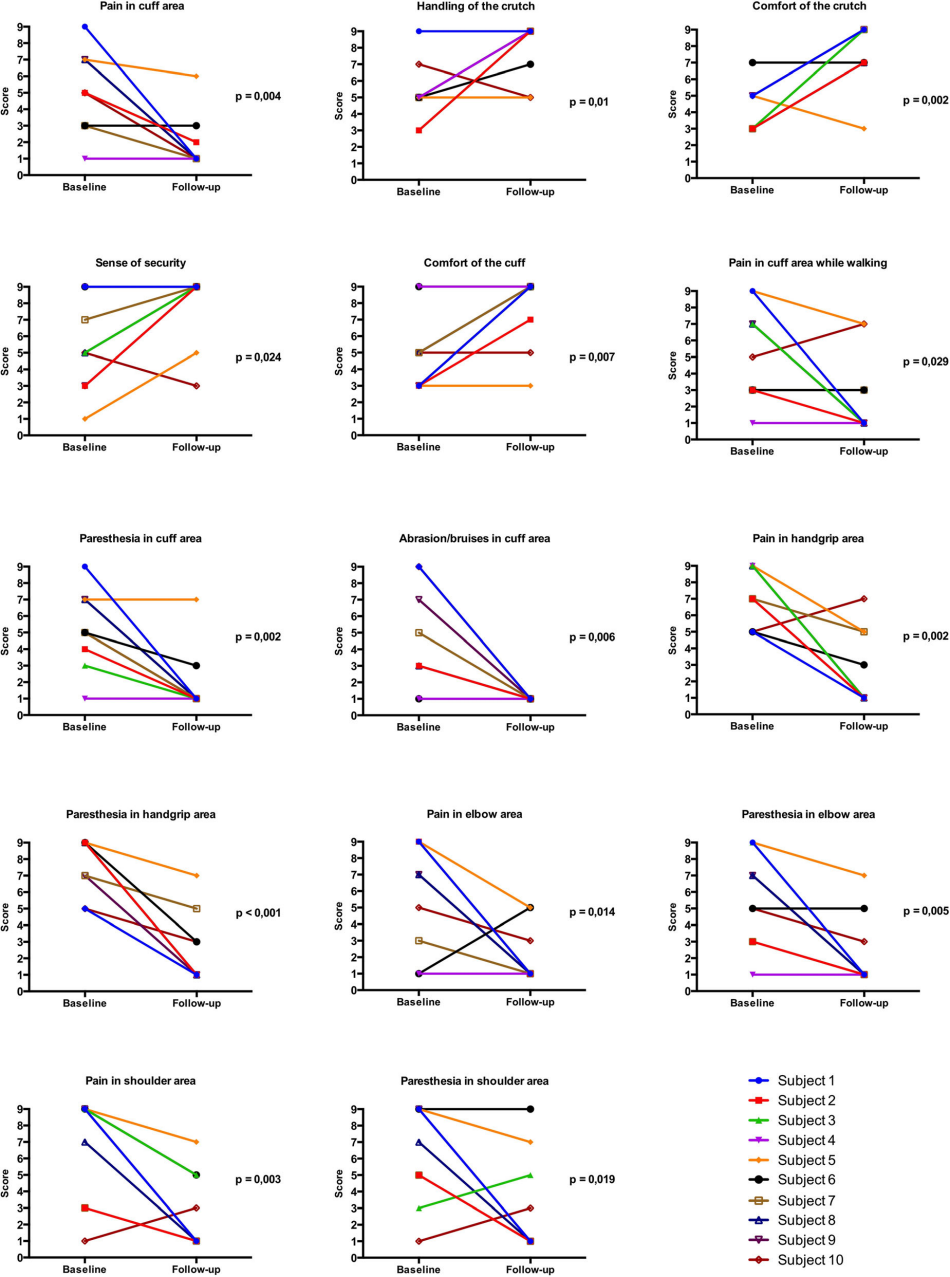
Individual change in scores of the UlnarPro questionnaires from baseline to 4-week follow-up for items 1 to 17

The mean pain score at the forearm decreased by 3.3 points (95% confidence interval difference (CI): [−5.0; −1.6]; Table 2; Fig. 3). Pain at the hands decreased on average by 4.6 points (95%CI: [−6.6; −2.8]). The two patients who had previously used anatomic hand grips also had lower pain at the hands at follow-up than at baseline. Pain at the elbows decreased by 3.6 points (95%CI: [−5.9; −1.3]; Fig. 3).

**Table 2:** Average (1 standard deviation) parameters describing pain, discomfort and function

Parameter	Baseline	4-week follow-up	*P*-value^1^
Pain			
Forearms	5.2 (2.4)	1.9 (1.6)	.004
Hands	7.2 (1.8)	2.6 (2.3)	.002
Elbows	5.6 (3.1)	2.0 (1.7)	.014
Discomfort			
Forearms	5.3 (2.3)	1.8 (1.9)	.001
Shoulders	6.6 (3.0)	3.0 (3.0)	.019
Hands	7.8 (1.7)	2.4 (2.1)	<.001
Elbows	5.8 (2.9)	2.2 (2.1)	.005
Skin bruise score	5.2 (3.4)	1.0 (0.0)	.006
Comfort			
General	4.2 (1.4)	7.6 (1.9)	.002
Forearm cuff	4.8 (2.4)	7.8 (2.2)	.007
Sense of security during walking	5.6 (2.8)	8.0 (2.2)	.024
Coping with daily routines	5.2 (1.8)	8.0 (1.7)	.010
Health			
General Health	26.0 (19.7)	37.0 (18.9)	.044
Physical health	20.0 (21.6)	36.9 (23.3)	.011
Bodily pain	26.8 (18.4)	42.5 (23.7)	.012

^1^ *P*-values of paired t-tests

### Discomfort and skin bruises

Discomfort decreased at the forearms by 3.5 points (95%CI: [−5.1; −1.9]), at the shoulders by 3.6 points (95%CI: [−6.1; −1.4],), at the hands by 5.4 points (95%CI: [−7.1; −3.7]) and at the elbows by 3.6 points (95%CI: [−5.5; −1.7]; Table 2; Fig. 3). The score for skin bruises also decreased significantly by 4.6 points (95%CI: [−6.6; −2.6]; Fig. 3). Three patients had a score of 9 for skin bruises at baseline and showed a decrease in this score to 1 at follow-up (Fig. 3).

### Comfort and sense of security

General crutch comfort significantly increased by 3.4 points (95%CI: [1.8; 5.0]; Table 2; Fig. 3), where comfort of the forearm cuff improved by 3.0 points (95%CI: [1.3; 4.7]; Fig. 3). The sense of security during crutch walking improved by 2.4 points (95%CI: [0.7; 4.1]; Fig. 3). Coping with daily routines improved by 2.8 points (95%CI: [1.1; 4.5]; Fig. 3).

### General health

**Fig. 4:**
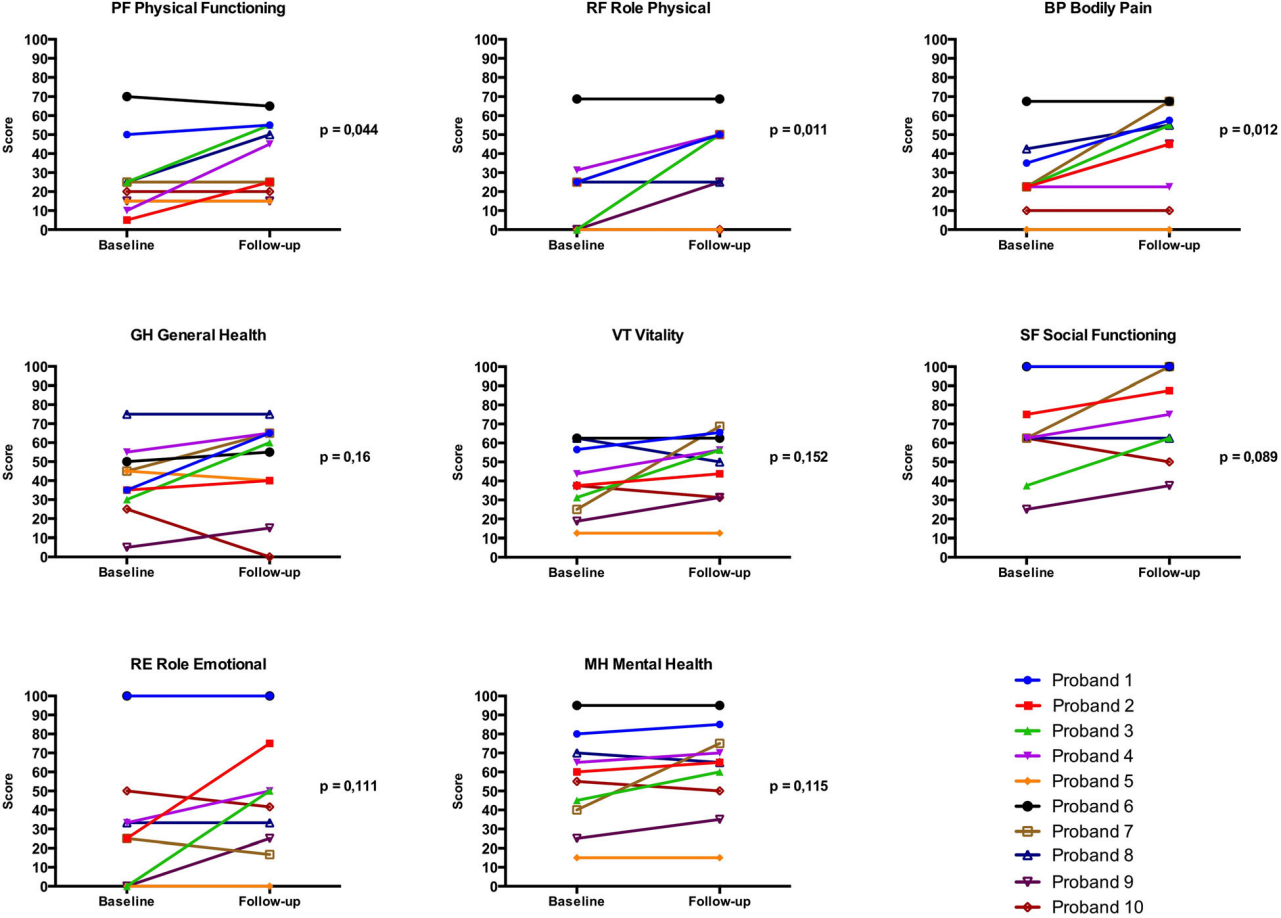
Individual change in scores of the different dimensions of the SF36 questionnaire from baseline to 4-week follow-up

We observed a significant increase in physical functioning by 11.0 points (95%CI: [1.8; 20.2]; Table 2; Fig. 4). The role physical score increased on average by 16.9 points (95%CI: [6.5; 27.2]) and bodily pain by 15.7 points (95%CI: [5.9; 25.6]). General health, vitality, social functioning, role emotional, and psychometric components of the SF36 questionnaire did not change between baseline and 4-week follow-up.

## Discussion

The purpose of this clinical pilot study was to determine if the use of forearm crutches with anatomic cuffs reduces pain and increases comfort and function in long-term users of forearm crutches during a 4-week period. The results of this pilot study clearly showed that compared to conventional crutches, pain and discomfort along the forearm significantly improve by the use of crutches with an anatomically shaped cuff. These results suggest that using an anatomically shaped crutch cuff is a promising solution to increasingly frequent clinical reports of pain and discomfort along the ulnar bone using conventional forearm walking aids especially in individuals with chronic walking disability.

Anatomic grips in forearm crutches have shown to reduce the load of the wrist, increase comfort and facilitate better control of crutch movements [10]. Although not measured in our study, an ulnar recess may have a similar effect by distributing the load during gait on the surrounding muscles and soft tissue. Comfort has been long identified to play an important role for devices interfering with human ambulation including footwear or braces. For instance, we have previously shown [24] that orthotic comfort is related to kinematics, kinetics and muscle activity. It appears feasible that greater comfort with forearm crutches may also alter ambulatory mechanics of crutch walking. Although measuring kinematics, kinetics or muscle activity during crutch walking was not within the scope of this paper, the results of this study may have important functional implications such as altered joint loads or walking efficiency. This possibility is further supported by the significant improvements in physical functioning in our study assessed using the SF-36 questionnaire.

The patient population included in this clinical pilot study was highly heterogenic in terms of age, underlying disorder and other variables. Most participants showed large improvements when using the anatomically shaped cuff despite of the heterogeneity in personal characteristics, and we were unable to identify patient subgroups responding differently to the new anatomic cuff. Nonetheless it is possible that older individuals with presumably less soft tissue who are more likely to develop nerve entrapment symptoms might benefit more from the ulnar protection. Moreover, it is conceivable that younger individuals may also experience and benefit from greater comfort with the anatomic cuff during mid- or short-term use of crutches. It should be noted, however, that correct instruction of crutch walking provided, for instance, by the physiotherapist is critical for preventing overuse symptoms [25–27].

This study was a clinical pilot study involving ten patients. The positive effect of the crutch with anatomic cuffs needs to be confirmed in a larger setting although blinding appears difficult because of the obvious differences between the anatomic and conventional cuffs. Eight patients had used conventional crutches with a normal grip prior to and hence also during the study whilst all crutches with anatomic cuff also had anatomic grips. Thus, potential effects of the anatomic grip on symptoms not only at the hands but also at the forearms cannot be excluded. However, changes in scores with the anatomic cuff did not depend on anatomic grip use. Interestingly, the two patients who had previously used anatomic grips also showed an improvement of their symptoms on the hands (Fig. 3, subjects 7 and 9). We therefore postulate that ulnar protection potentially may also have positive effects on the hands.

To date, evaluating specific aspects of forearm crutches has been difficult. In this study, we used a newly developed questionnaire comprising 17 questions categorized into four item categories. While this questionnaire has not been formally validated, cross-correlations among but not within item categories suggest that the item groups indeed assess the different aspects of pain and comfort of crutch walking investigated in this study. Hence, this questionnaire is very useful for future studies aimed at improving crutch design or for choosing optima crutches for a specific patient.

## Conclusion

The anatomic cuff for forearm crutches investigated in this study improved comfort and quality of life for patients with long-term crutch use. The significance of the anatomic cuff in reducing structural damage such as skin bruises or nerve entrapment should be investigated in larger trials.

## Abbreviation

OA: Osteoarthritis

## References

[1] Kaye HS, JKang T, LaPlante MP. Mobility device use in the United States. In: Research NIoDaR, editor. Disability Statistics Report 2000

[2] Vos T, Flaxman AD, Naghavi M, Lozano R, Michaud C, Ezzati M, Shibuya K, Salomon JA, Abdalla S, Aboyans V (2013). Years lived with disability (YLDs) for 1160 sequelae of 289 diseases and injuries 1990–2010: a systematic analysis for the global burden of disease study 2010. Lancet.

[3] Brown CJ, Flood KL (2013). Mobility limitation in the older patient: a clinical review. JAMA.

[4] Jones A, Silva PG, Silva AC, Colucci M, Tuffanin A, Jardim JR, Natour J (2012). Evaluation of immediate impact of cane use on energy expenditure during gait in patients with knee osteoarthritis. Gait Posture.

[5] Fisher SV, Patterson RP (1981). Energy cost of ambulation with crutches. Arch Phys Med Rehabil.

[6] Thys H, Willems PA, Saels P (1996). Energy cost, mechanical work and muscular efficiency in swing-through gait with elbow crutches. J Biomech.

[7] Krüger M, Bischof-Leger E (2008). Frequency of biceps tendon tenosynovitis from crutches: a sonographical observation. Z Rheumatol.

[8] Murphy MT, Journeaux SF (2006). Case reports: long thoracic nerve palsy after using a single axillary crutch. Clin Orthop Relat Res.

[9] Borrelli J, Haslach HW (2013). Experimental characterization of axillary/underarm interface pressure in swing-through crutch walking. J Rehabil Res Dev.

[10] Sala DA, Leva LM, Kummer FJ, Grant AD (1998). Crutch handle design: effect on palmar loads during ambulation. Arch Phys Med Rehabil.

[11] Goh JC, Toh SL, Bose K (1986). Biomechanical study on axillary crutches during single-leg swing-through gait. Prosthet Orthot Int.

[12] Ginanneschi F, Filippou G, Milani P, Biasella A, Rossi A (2009). Ulnar nerve compression neuropathy at Guyon’s canal caused by crutch walking: case report with ultrasonographic nerve imaging. Arch Phys Med Rehabil.

[13] Malkan DH (1992). Bilateral ulnar neuropraxia: a complication of elbow crutches. Injury.

[14] Garcia Suarez G, Garcia Garcia J, Perez CL (2001). Stress fracture of the ulna associated with crutch use. J Orthop Trauma.

[15] Fischer J, Nuesch C, Gopfert B, Mundermann A, Valderrabano V, Hugle T (2014). Forearm pressure distribution during ambulation with elbow crutches: a cross-sectional study. J Neuroeng Rehabil.

[16] Molteni P, Hugle T, Hugle M, Nuesch C, Mundermann A. Reduction in Ulnar Pressure Distribution When Walking with Forearm Crutches with a Novel Cuff Design: Cross-Sectional Intervention Study on the Biomechanical Efficacy of an Ulnar Recess. Assist Technol 2016. [Epub ahead of print] 10.1080/10400435.2016.1236045 27717292

[17] Canadian Red Cross. Guidelines on proper adjustment of forearm crutches. Available on http://www2.gov.bc.ca/assets/gov/people/seniors/health-safety/pdf/forearm_crutches.pdf, Accessed 20 Jan 2016.

[18] Cowley JA, Yongblood H (2009). Subjective response differences between visual analogue, ordinal and hybrid response scales.

[19] Bullinger M (1995). German translation and psychometric testing of the SF-36 health survey: preliminary results from the IQOLA project. International quality of life assessment. Soc Sci Med.

[20] Ware JE (2000). SF-36 health survey update. Spine (Phila Pa 1976).

[21] Royston P (1995). A remark on algorithm AS 181: the W-test for normality. J Roy Stat Soc Series C (Applied Statistics).

[22] Shapiro SS, Wilk MB (1965). An analysis of variance test for normality (complete samples). Biometrika.

[23] Sheskin DJ (2011). Handbook of parametric and nonparametric statistical procedures.

[24] Mündermann A, Nigg BM, Humble RN, Stefanyshyn DJ (2003). Orthotic comfort is related to kinematics, kinetics and EMG in recreational runners. Med Sci Sports Exerc.

[25] Lane PL, LeBlanc R (1990). Crutch walking. Orthop Nurs.

[26] Wu F, Ismaeel A, Siddiqi R (2011). Anterior interosseous nerve palsy following the use of elbow crutches. N Am J Med Sci.

[27] Van Hook FW, Demonbreun D, Weiss BD (2003). Ambulatory devices for chronic gait disorders in the elderly. Am Fam Physician.

